# A dendrite is a dendrite is a dendrite? Dendritic signal integration beyond the “antenna” model

**DOI:** 10.1007/s00424-024-03004-0

**Published:** 2024-08-09

**Authors:** Moritz Stingl, Andreas Draguhn, Martin Both

**Affiliations:** 1 Institute of Physiology and Pathophysiology, Medical Faculty, Heidelberg University, 69120 Heidelberg, Germany; 2 Department of Physiology, University of California, San Francisco, San Francisco, CA USA; 3 Neuroscience Graduate Program, University of California, San Francisco, San Francisco, CA USA

**Keywords:** Neurons, Neuronal physiology, Dendritic integration, Single-cell computations, Input–output relation, Review

## Abstract

Neurons in central nervous systems receive multiple synaptic inputs and transform them into a largely standardized output to their target cells—the action potential. A simplified model posits that synaptic signals are integrated by linear summation and passive propagation towards the axon initial segment, where the threshold for spike generation is either crossed or not. However, multiple lines of research during past decades have shown that signal integration in individual neurons is much more complex, with important functional consequences at the cellular, network, and behavioral-cognitive level. The interplay between concomitant excitatory and inhibitory postsynaptic potentials depends strongly on the relative timing and localization of the respective synapses. In addition, dendrites contain multiple voltage-dependent conductances, which allow scaling of postsynaptic potentials, non-linear input processing, and compartmentalization of signals. Together, these features enable a rich variety of single-neuron computations, including non-linear operations and synaptic plasticity. Hence, we have to revise over-simplified messages from textbooks and use simplified computational models like integrate-and-fire neurons with some caution. This concept article summarizes the most important mechanisms of dendritic integration and highlights some recent developments in the field.

## Introduction

**Fig. 1:**
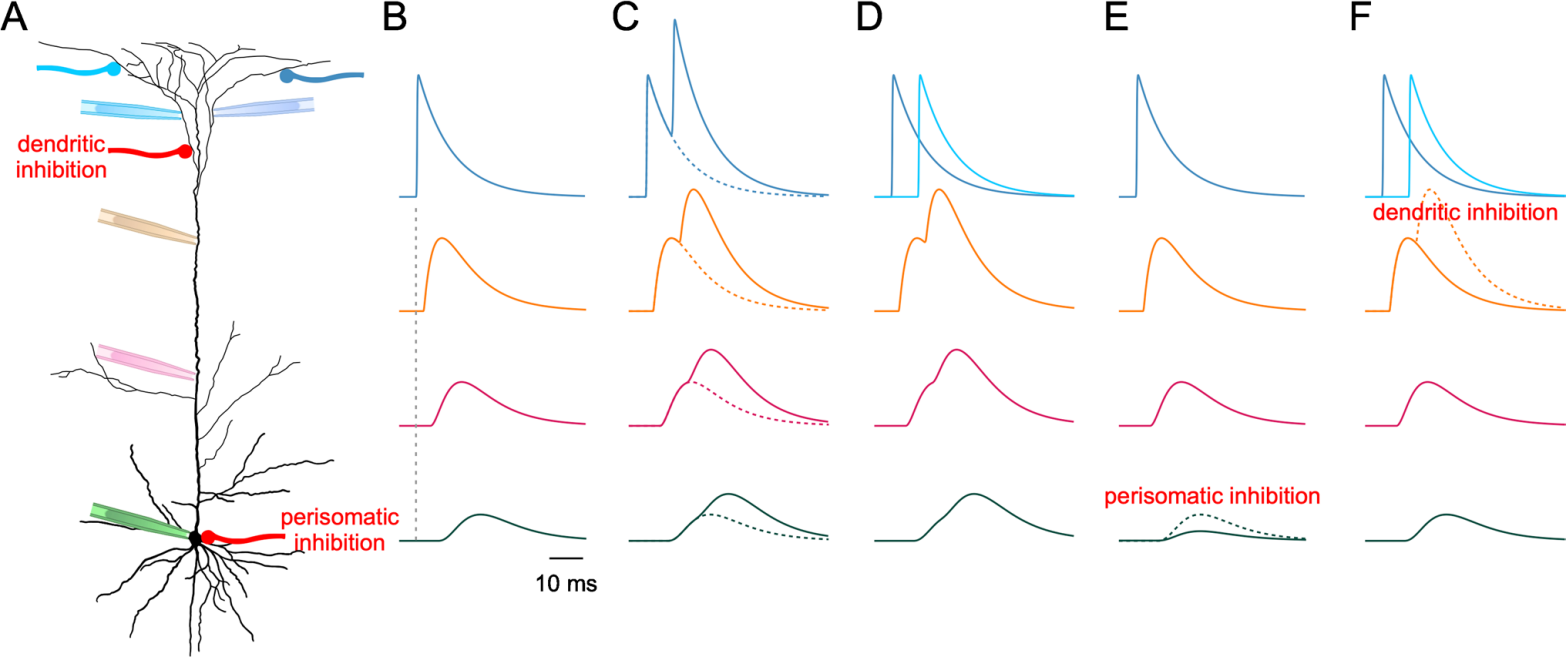
Passive summation beyond triviality.** A** Schematic representation of a cortical layer 5 pyramidal cell. Colored pipettes symbolize potential measurements at the soma (green), proximal (magenta) and distal (orange) stem dendrite, and two distal dendritic branches (blue). Dendritic or perisomatic inhibition, respectively, is indicated in red. **B** Attenuation of a peripherally generated EPSP during propagation towards the soma. Note decrease in amplitude and slowing of kinetics. **C** Generation of two EPSPs in the same dendritic branch. Simplified concept of integration as linear superposition of potential gradients. Again, there is a significant attenuation during dendritic-somatic propagation. **D** Generation of two EPSPs in different dendritic branches. Linear integration (simplified concept) occurs after confluence of peripheral dendrites in the common stem dendrite. Subsequent attenuation as in **B** and **C**. **E** Perisomatic inhibition reduces the impact of dendritic inputs at the somatic (and axonal) compartment, irrespective of its origin. **F** Inhibition in one dendritic branch can annihilate one input, but leave the other largely unchanged

Neurons are considered elementary units of information processing in neuronal networks, which, in turn, underlie behavior and cognition of all animals with central nervous systems. While neurons are, arguably, the most complexly shaped cells in all organisms, we often reduce them to highly simplified, point-like integrate-and-fire devices. In this textbook scheme, neurons collect multiple excitatory and inhibitory synaptic inputs at their dendrites. These potential transients propagate passively towards the soma and axon initial segment (AIS) and are linearly added wherever they superimpose (Fig. 1A–C). When the membrane at the AIS crosses a threshold value, the neuron produces an action potential, which triggers synaptic transmission to downstream neurons or effector cells. This simple model has inspired early concepts of neuronal computation, beginning with the seminal paper by McCulloch and Pitts [25], who showed that a network of nodes with elementary neuronal properties as above can perform basic logical operations (AND, OR, NOT). The McCulloch-Pitts neuron has enabled the subsequent development of artificial neuronal networks, which, meanwhile, use more complex, non-linear activation functions for their nodes. Modern artificial network architectures are still described using biologically inspired analogies, such as identifying nodes with neurons and edges with synaptic connections.

However, simplified connectionist models rely entirely on convergent and divergent connectivity and largely ignore the complexity of computations in biological neurons. Indeed, in most neurons, processing of synaptic inputs is all but linear and passive. Postsynaptic potentials are actively processed in all parts of the cell, most notably the dendrites [35]. Further, we begin to understand how the complex dendritic morphology, the strongly localized topography of excitatory and inhibitory inputs, and the intrinsic electrophysiological properties of neurons impact their computations. This field of research has become even more important since we are facing an exploding number of neuronal subtypes, which may all have unique mechanisms of signal integration [24, 34]. This short review shall summarize, in condensed form, the most prominent “active” properties of dendrites and highlight the resulting computational capacities of neurons. Many of our examples are taken from rodent cortical pyramidal neurons, which are of major importance for multiple cognitive processes and, at the same time, a well-studied model for dendritic integration [17, 24, 27]. We will not address the level of single dendritic spines, notwithstanding that these structures may constitute fascinating computational devices by themselves [11].

## Passive summation and propagation of synaptic potentials

We begin with a simplified model without active (voltage-dependent) dendritic conductances. Upon activation, a synapse stimulates a cluster of postsynaptic receptors, which transiently changes the local conductance of the postsynaptic membrane, usually leading to an ionic current. Synaptic currents mostly result in a local depolarization or hyperpolarization of the membrane, depending on the ion selectivity of the activated ion channels and the ion gradient across the postsynaptic membrane. If the local membrane potential happens to meet exactly the reversal potential of the activated ion channels, there will be no change in membrane potential. However, there is still an increased local membrane conductance for the duration of the synaptic activation, which causes a “shunting” (or short-circuit) effect for propagating potentials from other synapses. Neurons of central nervous systems typically receive thousands or tens of thousands of synapses. This notion implies that the contribution of most single synapses is small; otherwise, spontaneous synaptic activity would drive the neuron into constant, high-frequency action potential generation. Nevertheless, synaptic potentials are not negligible and exert a measurable effect both at their site of origin and, in most cases, at the site of action potential generation, usually the AIS.

How do locally generated postsynaptic potentials propagate to the soma and to the AIS? In a first approximation, dendrites can be compared to cables, which allow for passive propagation of electrical signals. Rall applied cable theory to synaptic signal propagation [30], showing that, along their way, synaptic potentials are strongly modified by the passive electrical properties of the dendritic “cable.” With its capacitance and resistance, the dendritic membrane forms a low-pass filter, which will inevitably slow down the kinetics of the original postsynaptic potential. Further, the longitudinal axial resistance of the cytosol-filled dendritic cylinder, together with dendritic leak conductances, attenuates the amplitude of propagating voltage signals (Fig. 1B). Thus, the time course of propagating postsynaptic potentials slows down, and the amplitude decreases on their way from the synapse to the AIS. This suggests that remotely located synapses are less efficient in triggering action potentials than those impinging more closely to the soma. However, while this notion is not entirely wrong, it is certainly a strong oversimplification. First, it appears that dendrites have an optimized thickness, tapering, and branch-point construction to reduce signal distortion through propagation as much as possible [6]. Second, in many neurons, the strength of synapses gradually scales up along the somato-dendritic axis [23]. Third, as we will see below, there are multiple active mechanisms compensating for the attenuation of remotely generated postsynaptic potentials.

Even in merely passive dendrites (if they do at all exist), the interaction of signals can be much more elaborate than a simple summation rule would suggest (Fig. 1C, D). First, the dendritic morphology of many neurons is specifically tailored to support their function. As a result of this, we can distinguish a vast diversity of neuronal cell types based on their uniquely shaped dendrites, which have been shown to affect, and sometimes even determine, how inputs are integrated. Especially in large neurons, the complex dendritic geometry enables the formation of loosely isolated compartments for electrophysiological activity and calcium dynamics [17, 35, 36]. Second, different synaptic input streams are often spatially ordered, such that afferent fibers of a specific origin target a specific portion of the dendrite. An example is dendrites of layer V cortical pyramidal cells, which receive association fibers from other cortical areas in their distal portion and thalamic input from specific sensory systems in more proximal dendritic regions. This spatial arrangement separates top-down (cortical) and bottom-up (sensory) information streams, which can be integrated in a controlled and state-dependent manner [16]. Third, practically all neurons are targeted by inhibitory synapses, which are also activated in a state- and behavior-dependent fashion. The dominant postsynaptic effect of these GABAergic or glycinergic inputs is an increase in postsynaptic chloride conductance, which counteracts excitation, usually by hyperpolarizing the membrane potential. Interaction with excitatory inputs can be complex, depending on the relative position and timing of the inhibitory and excitatory inputs, as well as the local transmembrane chloride gradient [14]. Importantly, specific populations of inhibitory (inter)neurons target different compartments. For example, (peri)somatic inhibitory synapses set the gain of the neuron’s input–output relation (Fig. 1E), inhibitory synapses at the axon initial segment tightly control action potential generation, and inhibitory synapses at defined dendritic segments attenuate the effects of layer-specific excitatory inputs (Fig. 1F) [39]. A more fine-grained analysis reveals an even larger diversity of inhibitory cells and mechanisms [15]. Given the rich variety and state-dependent activation of inhibitory interneurons in cortical networks, these cells are key players in the control of synaptic integration and in the organization of patterned network activity [40].

As an example of the intricate relation between neuronal structure and function, we have recently reported that in many hippocampal pyramidal neurons, the axon emerges from a basal dendrite, rather than from the soma [38]. When perisomatic inhibition is active, signal propagation from all dendritic branches to the axon is shunted, except from the axon-carrying dendrite where excitation reaches the AIS before encountering the inhibitory shunt. As a result, the axon-carrying dendrite is a privileged excitatory “input line,” especially in states with strong perisomatic inhibition. In such situations, neurons with axon-carrying dendrites are preferentially activated [13], and output from the local network is mostly restricted to this sub-group of pyramidal cells.

Together, these mechanisms show that “passive” summation in complexly shaped neurons does already allow for rich signal processing. However, this complexity is put to the extreme by active, voltage-dependent processes, which are present in most dendrites of central neurons.

## Active properties of dendrites

Dendrites are endowed with various species of voltage-dependent ion channels. Main “players” amongst these are voltage-gated sodium channels (VGSC), voltage-gated calcium channels (VGCC), NMDA-type glutamate receptors (NMDAR), and hyperpolarization-activated, cyclic nucleotide-gated (HCN) channels, together with different types of voltage-gated K + channels (VGKC) which mediate re-polarization [2, 7, 17]. These conductances are collectively termed “active,” as opposed to the “passive” redistribution of charges in cable-like structures as described above. Voltage-dependent mechanisms determine the waveform, frequency, and dynamics of action potentials. These “active intrinsic properties” of neurons are not only a defining feature of neuronal subtypes but also of utmost importance for their function within their respective networks. Important examples are state-dependent firing modes of thalamocortical neurons [19] and properties of intracortically projecting, regular spiking, and extracortically projecting, intrinsically bursting neocortical pyramidal neurons [24, 27]. The latter have been extensively studied with respect to dendritic signal processing [5, 17, 33] and serve as important examples in this review. In any case, active conductances support transformations of synaptic inputs far beyond the “passive antenna concept” of dendrites.

**Fig. 2:**
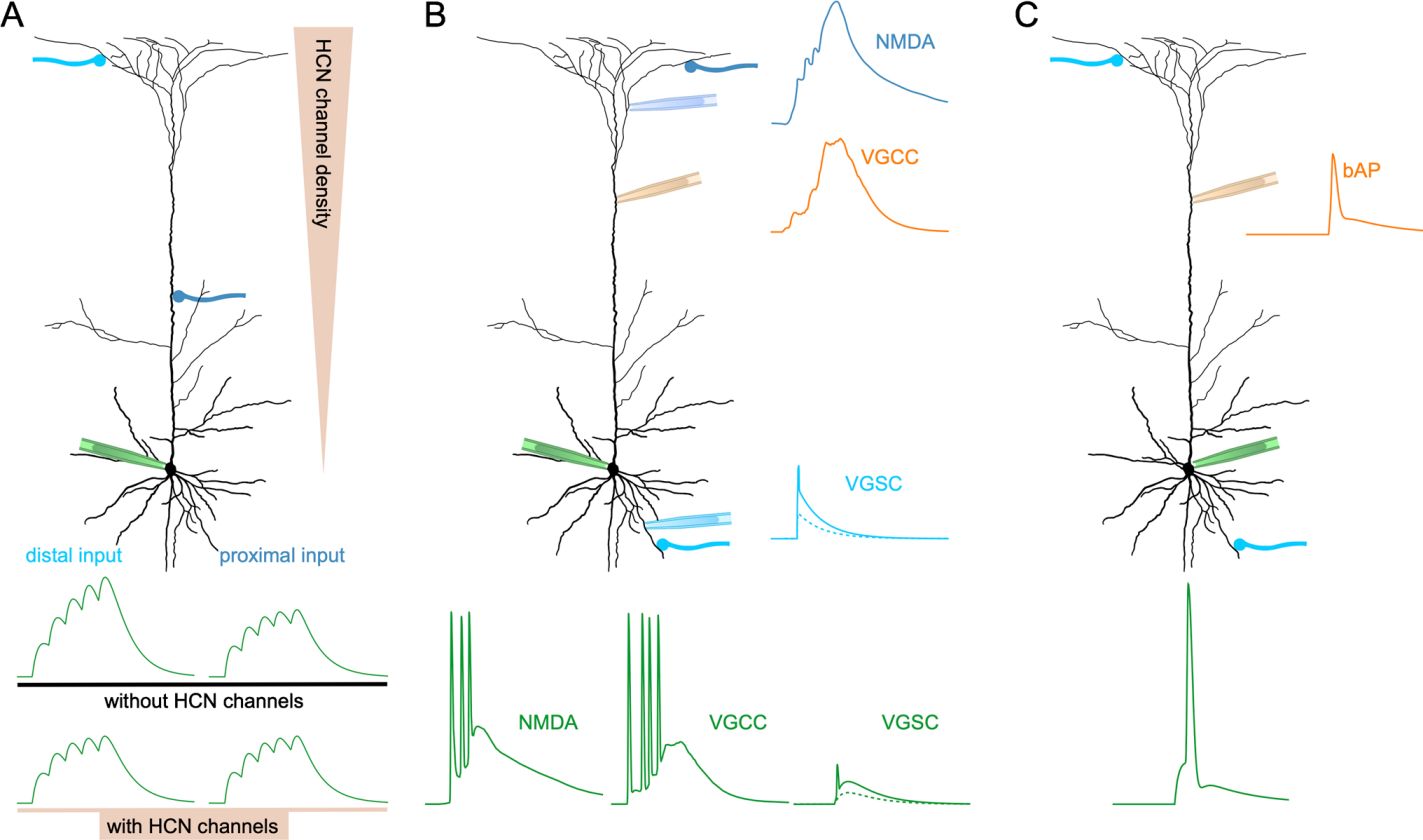
Effects of active conductances in dendrites.** A** Contribution of hyperpolarization-activated and cyclic nucleotide-gated ion channels (HCN channels). Integration of repetitive peripheral inputs is altered by the kinetics and voltage dependence of HCN leak conductances (see main text for details). **B** Activation of voltage-dependent ion channels increases and prolongs EPSPs, generating bursts (mediated by NMDA receptors or voltage-gated calcium channels) or partial spikes/spikelets at the somatic compartment. **C** Backpropagation of axonally generated action potentials into the dendrite. Simultaneous activation of excitatory synapses and bAP is a core mechanism of timing-dependent synaptic plasticity (see main text for details)

Active conductances can change the amplitude and waveform of propagating postsynaptic potentials. Due to the passive dendritic filter, the kinetics of EPSPs slow down as they travel to the soma (Fig. 1B). This effect becomes stronger the more distance an EPSP has to cover before reaching the AIS, which is why EPSPs generated in the distal dendrites will eventually be wider than EPSPs originating close to the soma. When multiple EPSPs occur in short succession, distally generated EPSPs will hence overlap more at the AIS due to their broader shape, resulting in increased temporal integration (Figs. 1C and 2A). However, many cortical neurons express cation-selective HCN channels in their dendrites. These channels are activated at negative membrane potentials and generate depolarizing currents, while they close when the membrane potential rises. With these properties, they increase dendritic membrane conductance at the resting potential, which increases amplitude loss of propagating EPSPs and supports the electrical compartmentalization of remote dendritic compartments [12]. When the dendritic membrane is depolarized, HCN channels close. This has two major effects: the membrane conductance decreases, limiting loss of amplitude during propagation of the EPSP along the membrane, and the loss of an inward current corresponds with an effective hyperpolarization, which shortens the apparent decay time course of the EPSP, thereby compensating for the low-pass filtering effect of the membrane (Fig. 2A) [2]. Interestingly, HCN channels are typically expressed with increasing density from soma to distal dendrites, which suggests that they systematically compensate or “normalize” for the kinetic distortion of remotely generated EPSPs [22].

However, normalization of inputs still emphasizes a soma-centric view on synaptic integration. In fact, information processing already begins in the dendritic compartment itself, with the generation of dendritic spikes (Fig. 2B) [17]. These are depolarizing voltage transients resulting from the activation of voltage-gated ion channels including VGCC, VGSC, and NMDAR. Just like their axonally generated namesakes, these conductances typically activate when a certain threshold potential is crossed, generating voltage transients with rather stereotypic waveforms. In most cases, the threshold for dendritic spike generation is only reached by coincident synaptic inputs to the respective dendritic compartment. The result is a strongly supra-linear EPSP summation: two inputs which, alone, would not reach the threshold for a dendritic spike can trigger a much larger, actively generated potential when they occur together. This dependence on synchrony of EPSPs is a key mechanism for the associative coupling of inputs. In short, dendritic spikes contribute to the role of neurons as “coincidence detectors” [10].

There is a great variety of dendritic spike waveforms and related mechanisms [17, 35]. We will briefly elude on the main types of voltage-dependent ion channels generating these voltage transients before summarizing their functional role for dendritic computation. Dendrites and somata of many neurons express VGSCs, though with lower density than axons [20]. With their typical fast kinetics, these channels can mediate steep potential fluctuations resembling classical action potentials (“dendritic sodium spikes”), but sometimes of smaller amplitude (“spikelets”). VGSCs support two types of active dendritic signals: peripherally generated spikes arising from EPSPs and backpropagating action potentials (bAPs). Peripherally generated spikes generally amplify local EPSPs, which may potentiate coincidence detection or counteract the attenuation of remotely generated signals (Fig. 2B). The other signal, bAPs, is a sometimes overlooked “by-product” of canonical action potentials generated in the AIS. Such spikes do not only travel down the axon but also invade the soma and “back”-propagate into the dendrites, where they can be amplified by activation of dendritic VGSCs, much like axonal action potentials during their active propagation (Fig. 2C). Depending on neuronal geometry, sodium channel density, and dendritic inhibition, bAPs may remain more or less confined to soma-near portions of the dendritic tree. Wherever they occur, they can boost coincident EPSPs and recruit NMDARs, which mediate a prolonged influx of calcium into the postsynaptic compartment. Thereby, bAPs are key for conversion of concomitant pre- and postsynaptic activity into lasting changes of synaptic strength. Dependent on the relative timing of pre- and postsynaptic neurons, this spike-timing-dependent synaptic plasticity can strengthen or weaken the synaptic connection [8].

NMDARs are mostly expressed in the dendrites and, as described above, have a firmly established role in plasticity and learning. These ion channels are activated by the binding of glutamate, but, due to the peculiar voltage dependence of these receptors, require a significant membrane depolarization before they open. Hence, their activity depends on an initial membrane depolarization provided by other sources than the NMDARs themselves, such as bAPs or the opening of nearby non-NMDA glutamate receptors. Because of these properties, NMDARs are coincidence receptors *sui generis*, and their activity makes a major contribution to the integration and amplification of coincident inputs. The spatial proximity of co-active synapses plays an important role for this mechanism: while coincident EPSPs originating in different dendrites may summate linearly at the soma, EPSPs occurring closely at the same dendritic branch can trigger NMDAR activation and add supra-linearly with a sigmoidal input–output curve [29]. Additionally, it has been shown that individual dendritic branches of cortical neurons can be sensitive to specific temporal sequences of synaptic activation. In such dendrites, the activation of more than three neighboring synapses in short succession leads to a particularly strong postsynaptic response, but only when the sequence starts at the periphery and progresses towards the soma. This spatiotemporal decoding depends on gradual changes of impedance along the dendrite, which results in asymmetric NMDAR activation based on the directionality of the input sequence [3]. Beyond a certain threshold, NMDAR activation can trigger large NMDA spikes, which usually occur in specific dendritic branches, e.g., in the basal dendrites of cortical pyramidal cells [32]. Together with voltage-gated calcium channels, NMDARs can elicit long-lasting plateau potentials in dendrites, which create a prolonged time window for boosting and integration of synaptic inputs (Fig. 2B) [17]. Recent evidence suggests that NMDAR-mediated dendritic spikes and subsequent increases in intra-dendritic calcium levels can remain confined to a local dendritic compartment [4], providing individual dendrites with the ability to regulate their gain independently from activity at the soma. Taken together, these mechanisms reinforce the concept of dendritic domains as semi-autonomous “computational subunits” that are capable of extensive signal processing and integration.

**Fig. 3:**
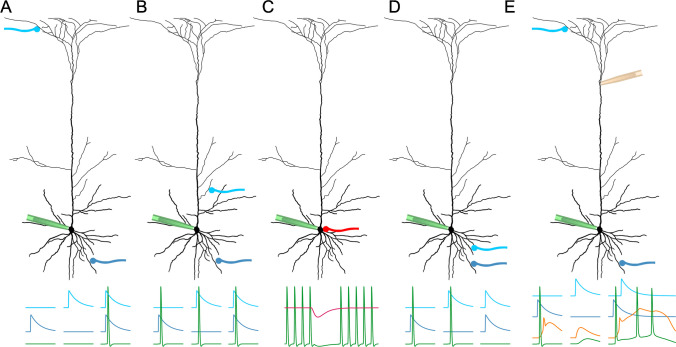
Information processing by dendrites.** A** Additive superposition of two weak excitatory inputs corresponds to a logical “AND.” **B** The effect of two strong excitatory inputs corresponds to a logical “OR.” **C** Perisomatic or axonal inhibition corresponds to a logical “NOT.” **D** Under certain conditions, spikes are only generated by either of two inputs, but not by their sum. This corresponds to a logical “XOR” (see main text for details). **E** Strong supra-linear superpositions of weak and strong inputs result from the activation of backpropagating action potentials and peripheral dendritic inputs (modified from [16])

Finally, dendritic spikes mediated by VGCCs occur in all dendritic compartments. L-type calcium channels are, for example, prominently expressed in the apical dendrite of layer 5 pyramidal cells, where they may enhance synaptic input by interacting with bAPs [18]. This discovery was of particular importance, because it was previously unclear how inputs to the distal dendrites, or even locally constrained distal dendritic calcium spikes, would exert strong effects at the soma or AIS. However, the pairing of dendritic EPSPs with a single bAP can lead to the generation of a strong dendritic calcium spike, which propagates to the AIS and typically triggers a burst of action potentials (Fig. 3E). Like NMDAR-mediated spikes, VGCCs can give rise to dendritic plateau potentials lasting tens of milliseconds or more (Fig. 2B). These potentials may be of particular importance to pre-depolarize the dendrite and make the neuron more excitable in response to subsequent synaptic activity. Plateau potentials can mediate interactions between different neuronal compartments (especially distal dendrites and axo-somatic compartments) or coincident, but not strictly synchronized synaptic input streams [17]. In cortical pyramidal neurons, they might support the integration of distal, associative dendritic input streams and more proximal, specific thalamic inputs from sensory systems. This may relate to the processing of top-down (cortical) and bottom-up (sensory) information and contribute to attention, perception, and even consciousness [16, 17, 37].

While we have focused on three different ion channel families mediating depolarizing voltage shifts, it should be mentioned that potassium-mediated hyperpolarizing currents are required for re-installing resting conditions. The kinetics of this re-polarization depends strongly on the type and density of voltage-gated potassium channels in the different dendritic compartments [7]. An intriguing example of interaction between dendritic spikes and specific potassium channels is the downregulation of Kv4.2 channels by NMDA receptor-mediated spikes in the respective dendritic compartment [21], which in turn increases the coupling between axo-somatic and dendritic spikes.

## Conclusion: dendrites as complex computational devices

The above-described mechanisms show how the spatial complexity and compartmentalization of neurons are reflected in their rich computational repertoire. Conventional action potential generation in the AIS is just the final step in a cascade of non-linear operations. Much of the synaptic signal processing has actually been done in the dendrites before synaptic potentials reach the axo-somatic compartment, and much dendritic computation is affected by backpropagation of axonally generated action potentials into the dendrites. Thus, dendrites are computational devices in their own right.

To illustrate the computational power of dendrites, we come back to the initial remarks on McCulloch and Pitts [25]. The first artificial neuronal networks (perceptrons, [31]) could reproduce logical functions like AND, OR, and NOT, but were unable to reproduce the exclusive OR (XOR) function, where an output value of “1” is generated when one of two inputs is “1”, but not when both of them or none is “1” [26]. This limitation could only be overcome with more complex networks comprising more than a single input and a single output layer. It is relatively simple to see parallels between the logical operations of AND, OR, and NOT on one hand, and synaptic summation or inhibition processes in biological neurons on the other (Fig. 3A–C). In contrast, the XOR problem was long thought to require more complex physical structures, similar to a multi-layer artificial neuronal network. However, a recently discovered mechanism reveals how dendritic calcium spikes in human cortical pyramidal neurons do actually reproduce the logical XOR operation: they generate high amplitudes and reliable dendritic-somatic coupling when they are elicited by either one of two groups of synaptic inputs, but decrease in amplitude or vanish when both input streams are simultaneously activated (Fig. 3D) [9]. This finding is an impressive example of the richness of logical operations, which non-linear dendritic integration mechanisms can achieve. To capture this complexity, recent computer models represent single neurons as multi-layer, deep artificial networks [1]. This complexity challenges the traditional equation of neurons with simple “integrate-and-fire” nodes and necessitates new theoretical approaches to understand the contribution of single neurons to network- and system-level operations underlying behavior and cognition [28]. Experimentally, uncovering the full potential of dendritic signal processing will require a large body of work using high-resolution methods like electrophysiological recording from dendrites, high-speed calcium and sodium imaging in vitro and in vivo, and high-resolution reconstructions of dendritic morphology.

When we extrapolate these challenges to the vast variety of different neuronal subtypes, functional states, and cognitive-behavioral contexts under study, we can safely say that understanding single-neuron computation will keep us busy for the coming years. The old textbook paragraphs on linear summation of synaptic inputs cover merely the least complex of the multiple operations which dendrites can perform.

## Data Availability

No datasets were generated or analysed during the current study.
